# You are how you recruit: a cohort and randomized controlled trial of recruitment strategies

**DOI:** 10.1186/1471-2288-14-111

**Published:** 2014-09-27

**Authors:** Amy Maghera, Paul Kahlke, Amanda Lau, Yiye Zeng, Chris Hoskins, Tom Corbett, Donna Manca, Thierry Lacaze-Masmonteil, Denise Hemmings, Piush Mandhane

**Affiliations:** Department of Pediatrics, University of Alberta, Edmonton, Alberta Canada; Department of Family Medicine, University of Alberta, Edmonton, Alberta Canada; Department of Pediatrics, University of Ottawa, Ottawa, Ontario Canada; Department of Obstetrics and Gynaecology, University of Alberta, Edmonton, Alberta Canada; Respiratory Medicine; Department of Pediatrics, University of Alberta, 4-590 Edmonton Clinic Health Academy (ECHA), 11405 87 Avenue, Edmonton, AB T6G 1C9 Canada

**Keywords:** Recruitment, Birth cohort, Research methods, Sample bias

## Abstract

**Background:**

Recruitment is a challenge in developing population-representative pregnancy and birth cohorts.

**Methods:**

We developed a collaborative recruitment infrastructure (CRI) to recruit pregnant women for 4 pregnancy cohorts using: faxes from obstetrical offices, in-clinic recruiters, university and funder-driven free-media events, paid-media, and attendance at relevant tradeshows. Recruitment rates and demographic differences were compared between recruitment methods.

**Results:**

We received 5008 referrals over 40 months. Compared to fax, free-media referrals were 13 times more likely to be recruited (OR 13.0, 95% CI 4.2, 40.4: p < 0.001) and paid-media referrals were 4 times more likely to be recruited (OR 4.6, 95% CI 2.1, 10.3: p < 0.001). Among paid-media advertisements, free-to-read print (e.g. Metro) was the most effective (OR 3.3, 95% CI 2.3, 4.5: p < 0.05). Several demographic differences were identified between recruitment methods and against a reference population. Between recruitment methods, media recruits had a similar proportion families with incomes ≥ $40,000 (paid-media: 94.4%; free-media: 93.3%) compared to fax recruits (95.7%), while in-clinic recruits were less likely to have family incomes ≥ $40,000 (88.8%, p < 0.05). Maternal recruits from fax and in-clinic were more likely to attend university (Fax: 92.6%, in-clinic 89.8%) versus the reference population (52.0%; p < 0.05 for both) and both were less likely to smoke (Fax: 6.8%, in-clinic 4.2%) versus reference (18.6%; p < 0.05 for both). However, while fax referrals were more likely to be Caucasian (85.9% versus reference 77.5%; p < 0.05), in-clinic referrals were not significantly different (78.2%; P > 0.05).

**Conclusion:**

Recruitment methods result in different recruitment rates and participant demographics. A variety of methods are required to recruit a generalizable sample.

## Background

There is a renewed interest in large, population-representative pregnancy and birth cohort studies. The UK government recently announced a 90,000 longitudinal pregnancy/birth cohort project [1]. The USA National Children’s Study (NCS) plans to recruit 100,000 children [2]. One of the biggest challenges in developing these studies is participant recruitment [3, 4].

Although the NCS originally proposed a pre-conception cohort and estimated that 10–40 households would need to be approached for every enrolled birth, the yield has been markedly lower: 163 households/pregnant women recruited. The NCS is now developing a more traditional pregnancy/birth cohort with recruitment from prenatal care sites [5]. Cohort studies such as the Right from The Start Study (RFTS) [6] and Avon Longitudinal Study of Parents and Children (ALSPAC) Study [7] highlight the need for a multifaceted recruitment approach (i.e. community recruitment, prenatal clinics, advertising) with cooperation from collaborators. The NCS’s recently proposed using three recruitment strategies: hospitals and birthing centres, physician/provider referrals, and targeted recruitment for participants of particular scientific interest (i.e. those affected by health disparities, lack of health care access). The NCS intends to recruit a fixed number of participants from each strategy (45000/45000/10000) [8]. We hypothesized that the different recruitment methods will have different recruitment rates, and each recruitment method will approach and recruit a slightly different demographic of participants. Between 2008 and 2012, there were four pregnancy cohort studies recruiting at the University of Alberta. We developed a collaborative recruitment infrastructure (CRI) that recruited for all four studies simultaneously. In this paper we present the results of the CRI.

## Methods

### Description of participating studies

#### Alberta Pregnancy Outcomes and Nutrition (APrON)

APrON is a study involving thousands of women from Alberta designed to analyze the relationship between maternal nutrient status during pregnancy, maternal mental health, and child health and development. The purpose of APrON is to determine the impact of maternal nutrient intake and status on their own mental health and their children’s neurodevelopment and mental health http://www.apronstudy.ca.

#### Canadian Healthy Infant and Longitudinal Development (CHILD) study

CHILD is a pan-Canadian longitudinal birth cohort study of 3500 children with follow-up until five years of age. The purpose of the CHILD study is to determine what aspects of the environment interact with genetic factors to affect children’s health and development with a focus on the development of atopic diseases such as asthma. The study includes multiple health and environmental assessments at frequent intervals throughout the study http://www.canadianchildstudy.ca.

#### Maternal-Infant Research on Environmental Chemicals (MIREC)

MIREC aims to recruit approximately 2000 women from 10 sites across Canada. There are 3 main objectives for MIREC: to measure the extent to which pregnant women and their infants are exposed to chemicals; to measure some of the beneficial elements in human breast milk; and to assess what health risks, if any, are associated with the chemical levels measured, with a focus on heavy metals such as lead and mercury. http://www.mirec-canada.ca.

#### Trauma in Pregnancy study (TIPS)

TIPS investigates the effects of subtle stressors, as well as of more severe, obvious traumas, on child development and maternal health. Participants are divided into a case study group of 120 women who have experienced trauma during pregnancy and a control group of 120 women whose pregnancies are low risk and trauma free. Mothers are assessed every two months postpartum, and babies are assessed at 6, 12, and 18 months after delivery.

### Recruitment methods

The University of Alberta Health Research Ethics Board (HREB) approved the CRI globally and for each participating study separately. The general procedure for recruitment is as follows. Prospective participants had nominal information collected to determine their eligibility for recruiting studies. Study specific Research Assistants (RAs) contacted the prospective participants, now called referrals, to determine their interest in participanting in one of the studies. If a referral was interested, they would be consented to their respective study. After consenting, the referrals were called recruits. Written informed consent was obtained from the participants for the publication of this manuscript and any accompying images. A copy of written consents are available for review by the Editor of this journal.

The CRI used six methods to obtain referrals and recruit pregnant women. Initially we used a single pamphlet to present all studies for the fax and free-media methods. We subsequently chose to distribute study specific pamphlets on a rotating basis.

**Figure 1:**
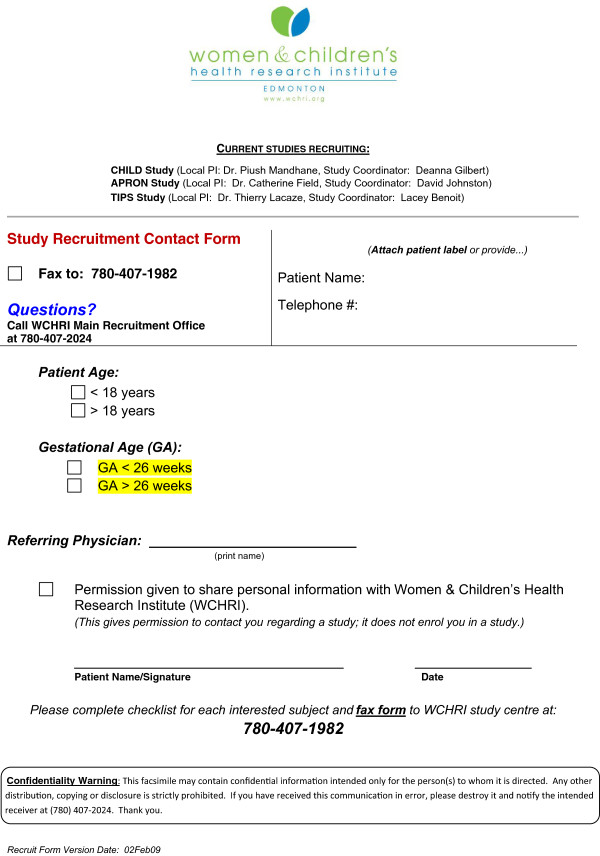
**Fax recruitment screening form (compressed).**

- *Fax:* The fax method was developed around the observation that study endorsement by a patient’s health-care provider is an important determinant of recruitment [9, 10]. During pre-natal visits, the clinic’s front desk staff would provide a study pamphlet for the patient to read while waiting for her appointment. The clinic staff (physicians, mid-wives, nurses) would then take up to 1 minute to present the study(s) and ask their patient whether she would consent to having her contact information faxed to the CRI office (fax sheet: Figure  1). Faxes were distributed to the studies that were concurrently recruiting based on nominal inclusion and exclusion criteria. RAs subsequently contacted the women to determine if she would be willing to participate. We remunerated each group practice, minimum 3 physicians with a focus on obstetrics, participating in the fax or in-clinic method (see following) $250/month. We maintained an active dialogue with participating physicians by regular visits and distributing a CRI newsletter.
- *In-clinic:* We stationed RAs in the waiting room of some high volume obstetric clinics. The RAs from the different recruiting studies rotated among the clinics and presented their study to prospective participants. The in-clinic RA had up to 2 minutes to present the study and included some of the personal benefits to participation - the potential for personal benefit is the number one reason why pregnant women participate in research [9, 10].
- *Tradeshows:* APrON and CHILD each had booths at several pregnancy or infant tradeshows. Both studies also held information nights at community centers. Study material was presented in addition to providing study-marketing material such as reusable bags and pens. CHILD subsequently instituted a gift basket draw with ballots available at the tradeshows. The raffle was free and all individuals (pregnant or not) were eligible.
- *Free-media:* Participant interest that resulted from free recruitment methods (i.e. media interest, brochures and poster in locations without RA study endorsement) [11].
- Separate press releases for CHILD and APrON resulted in study coverage in local and national media.
- Posters and brochures were placed in locations that provide services for pregnant women. Locations included:
- Small volume obstetrics
- Family physician clinics
- Phlebotomy laboratories
- iv. Birth control centers
- Pre-natal ultrasound imaging centers
- Maternity and baby stores

5. *Direct:* Prospective participants would call in to the CRI study office or the study-specific office based on recommendations from friends or other physicians.

**Table 1:** **Advertisement RCT details**

Advertisement intervention	Media outlet	Audience/circulation	Demographics (if available)	Publishing frequency	Advertisement type/duration	Number of interventions	Wash-out period	Total cost
Postal	Canada Post			Daily	2500 flyers	1	1 week	$451.50
Free-to-read print	Examiner	141000	28,000	Weekly	2 weeks	1	1 month	$2981.60
			18-39 Females/day					
	See	24000	36% 25-34 yr	Weekly	2 weeks	1	1 month	
	Metro	68000	Mostly young adult	Daily	2 days	1	1 month	
Transit	Interior bus cards			1 month	50 cards	2	1 month	$2300
	Train Poster			1 month	1 poster	2	1 month	
	Back of the bus poster			1 month	1 poster	1	1 month	
Paid-to-read print	Sun	129000	36% 18-34	Daily	¼ pg. color	2	1 week	$5080
	Journal	269000	48% 18-49 y.	Daily	¼ pg. color	2	1 week	
Birth Issues	Birth Issues			Quarterly	1	4	N/A	$800
Trade-specific publications	Today’s Parent			Monthly	1 month	2	1 month	$5607
	Edmonton Woman			Bimonthly	2 months	2	2 month	
	Edmonton Child			Bimonthly	2 months	2	2 month	
Internet	See.ca / Vue.ca	1000 impressions		Daily	1 month	1	1 month	$2950
	CBC.ca			Daily	1 month	2	1 month	
	Facebook advertising		F 18-39	Daily	10 click-through/day	14	1 month	
Radio	CISN: #3 Radio	63,000	F 18-42	Weekly	1 month	2	1 month	$5760
	CISN online			Weekly	1 month	2	1 month	
	Joe: #10 Radio	75,000	F 18-42	Weekly	1 month	2	1 month	
	Joe Online			2 weeks	1 month	2	1 month

6. *Paid-media:* Between November 1, 2010 and January 30, 2012, the CHILD study completed a randomized (random blocks) control trial (RCT) to determine the effectiveness of paid-media advertising on study recruitment. Our total advertisement budget was $25 000. The choice of advertisement interventions was determined by discussions with senior principal investigators (who suggested radio), the experience of several senior research coordinators (who suggested transit and free-to-read print), and limited budget (e.g. internet, Facebook, postal). The choice of media outlet (e.g. Sun, Metro, Today’s Parent, CBC.ca, etc.) within each intervention was chosen based on the media outlet demographics. The duration of each advertisement within each media outlet was based on budget considerations in consultation with the media outlet vendor around the optimal duration for the advertisement. Advertisements (e.g. radio script, print advertisement pictures, and logos) were developed by a professional advertisement agency in consultation with the media outlet. Table  1 provides the details for each of advertisement interventions. Advertisement interventions included:

**Figure 2:**
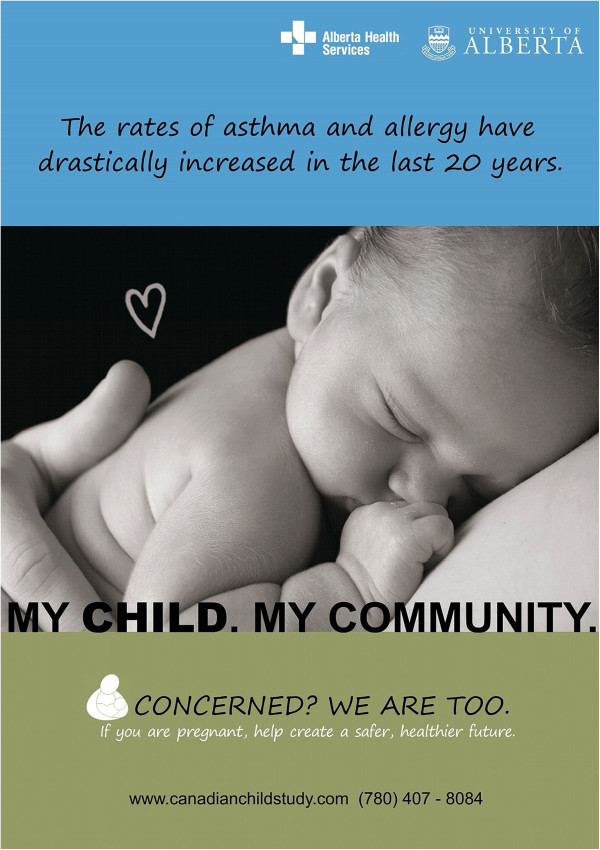
**Example of free-to-read print advertisements.**

**Figure 3:**
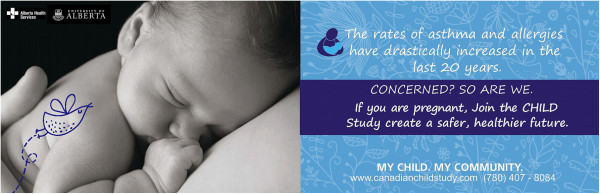
**Example of the trade-specific publication advertisements.**

**Figure 4:**
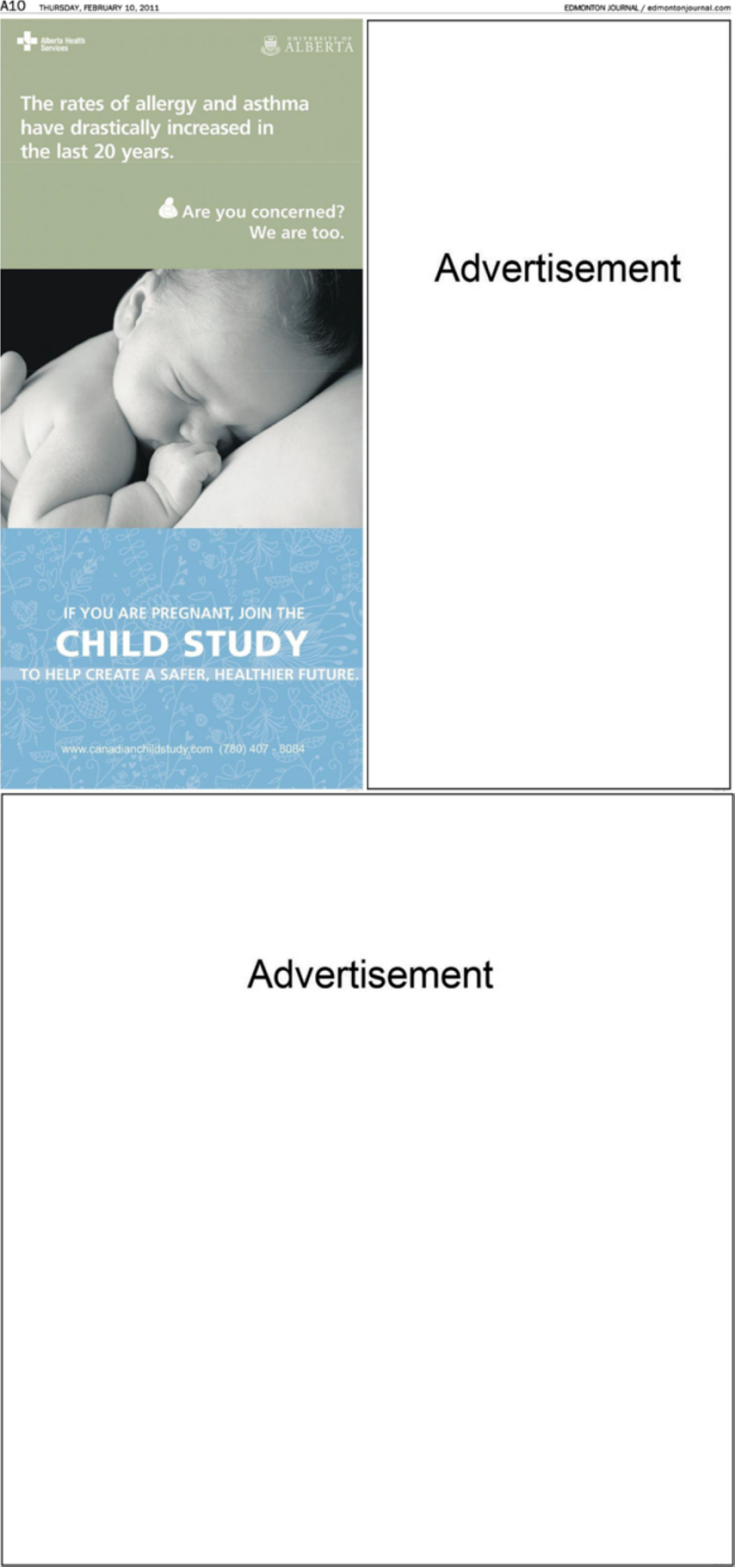
**Example of paid-to-read print advertisement.**

**Figure 5:**
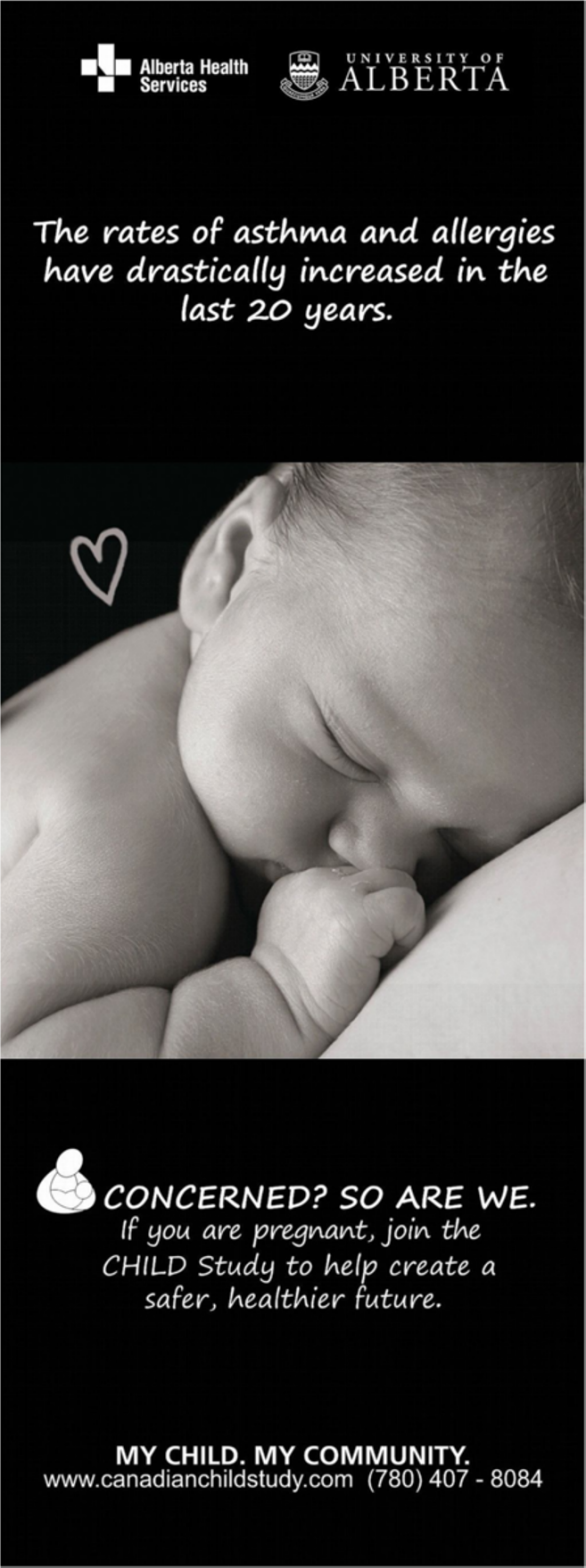
**Birth Issues Ad.**

- Postal (e.g. Advertising mail-out flyer to a community with a high birth-rate)b. Free-to-Read Print (e.g. Metro, Examiner; (Figure  2))
- Transit (e.g. Buses, trains, and bus benches)d. Trade-Specific Publications (Figure  3)e. Paid-to-Read Print (e.g. Edmonton Journal (Figure  4), Edmonton Sun)
- Internet (e.g. Facebook, CBC.ca, See.ca)
- RadioThe order of advertisement intervention was randomly determined (random number). A washout period between interventions of at least 4 times the duration of the intervention was included after each intervention. One of the interventions (Birth Issues) has a 3-month publication cycle. Inclusion of Birth Issues in the RCT would necessitate a 1-year wash-out period. A 1-year wash out would not allow us to complete the advertisement RCT prior to the end of recruitment for the CHILD study. As a result, we elected to advertise in Birth Issues for 1 year during the advertisement RCT (Figure  5).

### Tracking referrals through the CRI

A Research Electronic Data Capture: RedCap [12] database was used to input, assign, and track referral and recruits. The database records basic contact information (name and telephone number) and gestational age (GA) ≤24 weeks. Also, the database logs the date and time of each contact attempt, the results of the attempt (e.g. contacted, answering machine, message left), and any conversation results (e.g. recruited, declined).

### Statistical analyses

#### Contacting individuals

We examined the number of calls required by each recruitment method to reach a resolution (recruited, ineligible, or declined.) Generalized linear mixed model with robust errors was used to examine the influence of factors such as such as time of day and day of week of the call on the likelihood of talking to an individual on the phone.

#### Recruiting individuals

Univariate and multivariate logistic regression (Stata9.2; Stata Corp, College Station, TX) compared the different recruitment methods for the odds of recruiting individuals, while controlling for recruiting study and recruit demographics. Fax recruitment/recruits were used as the reference group because 1) fax recruitment is analogous to the physician referral recruitment strategy most commonly used by research studies, 2) had the largest sample size and 3) fax was the first recruitment strategy utilized.

For both free and paid-media, we analyzed (Poisson regression for count data) for the number of individuals who reported being recruited as a results of a specific advertisement (media-specific recruits) and for all individuals recruited during each advertising intervention as a reflection of increased general awareness of the CHILD study (multi-level Poisson regression controlling for the specific clinics for the fax and in-clinic referrals). We considered a lag between 1 to 4 days in the models (using auto-correlation) to capture the delay between advertising and an individual’s response to the advertisement. For each advertising intervention we included referrals received up to 2 days after the last advertisement as part of the intervention based on the auto-correlation results. We included the presence of Birth Issues advertising in all analyses that included paid-media.

#### Demographic differences between referrals

Univariate and multivariate logistic and linear regression compared recruitment methods for the age of referrals (calculated from birth date where available), proportion ≥24 weeks GA, ethnicity as determined from last names (general Canadian population, South Asian origin, Chinese origin) [13], and income quintile (imputed from the 6-digit postal code using the Statistics Canada data Postal Code Conversion File (PCCF+)) [14].

#### Demographic differences between recruits

CHILD study data was used to examine differences in the recruit’s demographic data by recruitment method. Univariate and multivariate logistic and linear regression compared recruits by income strata, country of birth, health conditions, mother’s age, and proportion of mothers ≥24 weeks GA at time of recruitment.

The demographics of those recruited to the CHILD study were compared to a reference population of pregnant women identified through a multiple-physician obstetrical practice in Edmonton [15]. Univariate analysis compared recruits (total and by recruitment method) to the reference population for marital status, ethnicity, education, income strata, and health conditions.

## Results

**Figure 6:**
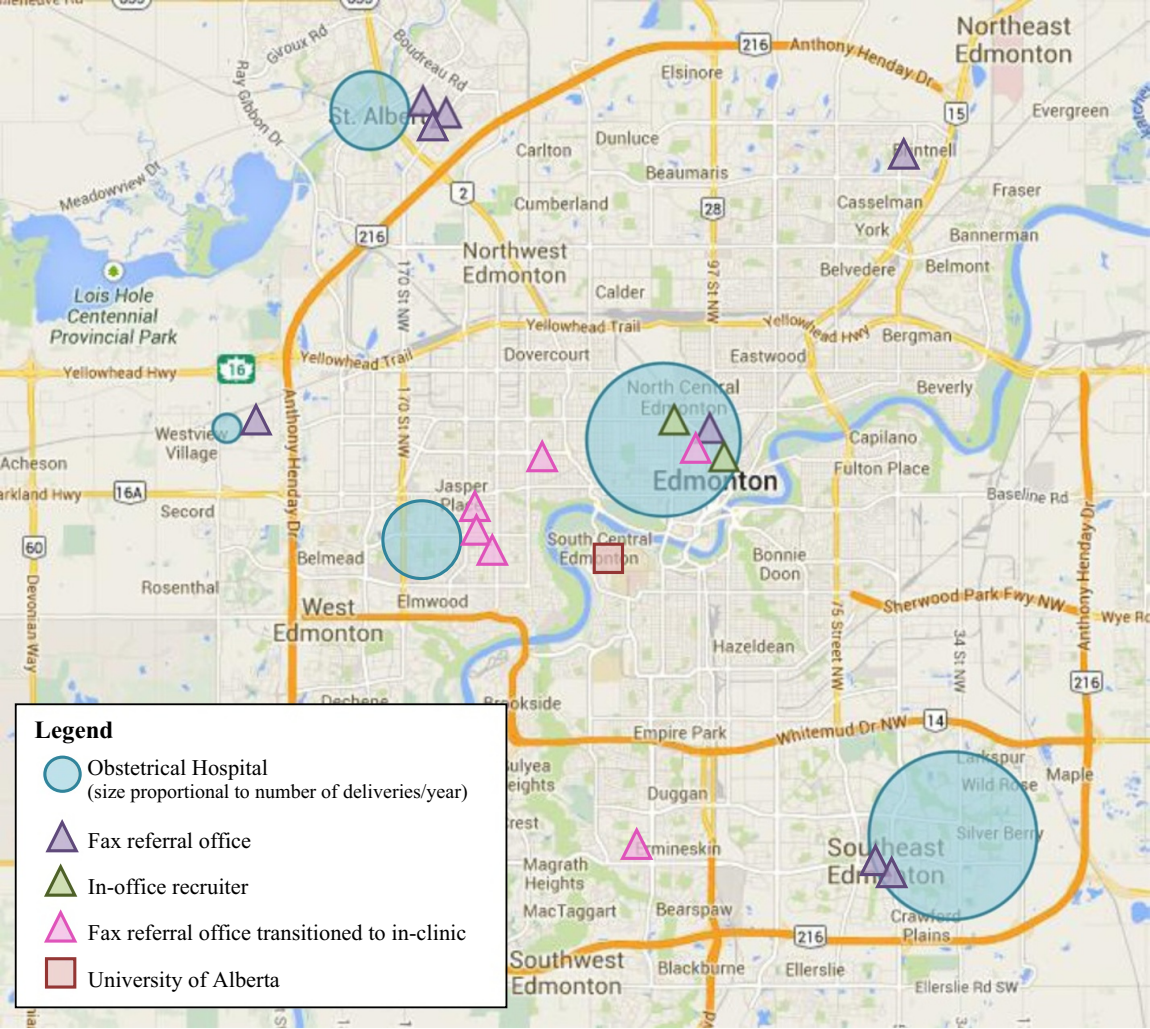
**Location of obstetrical hospitals, fax, and in-clinic recruiters.**

**Figure 7:**
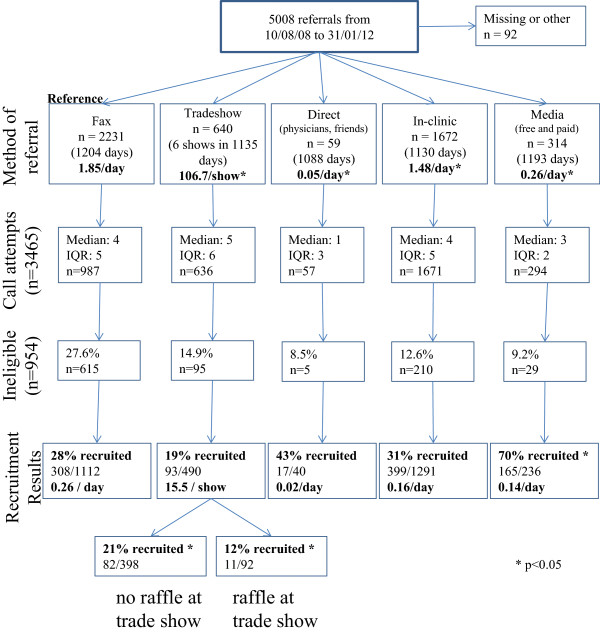
**Referral and recruitment results by recruitment method.** Legend: *p < 0.05.

The CRI was implemented at sixteen sites across Edmonton (Figure 6). We received 5008 referrals from 10/03/08 to 31/01/12 (Figure 7). There were significant difference in referral rates between fax (reference group) and all other recruitment methods for all studies. We received the most referrals through fax (1.85 referrals/day) and the least from the direct recruitment method (0.05 referrals/day; p < 0.05). Each tradeshow garnered approximately 46 referrals (p < 0.05 versus fax referrals).

From the 3465 referrals we have call data on, individuals were more likely to be spoken to in the evenings (40.3%; p < 0.001; Table 2) compared to mornings (34.0%) and afternoons (35.2%). Individuals were also more likely to be contacted on the weekend (Saturday: (Odd ratio (OR): 1.68; 95% CI 1.06, 2.66; p: 0.03; Sunday: OR 1.52; 95% CI 0.95, 2.42 p: 0.08) compared to weekday calls. Referrals identified through direct recruitment required the fewest calls (Median 1 call, Interquartile range (IQR) 3) to reach resolution (recruited or declined), followed by media (Median 3 calls, IQR 2), in-clinic and fax (Both: median 4 calls; IQR 5), and tradeshows (median 5 calls, IQR 6). Compared to fax, call attempts from all other recruitment methods were statistically significantly different (p < 0.001).

**Table 2:** **Chance of talking to someone by time of day, day of week, and by gender of RA caller at the date of the call (n = 16092 data points)**

	Percentage	OR (95% Conf. interval)	p-value
**Time of day**			
Morning (09:00 – 12:59)	34.0% (1698/4992)	Reference	
Afternoon (13:00 – 16:59)	35.2% (3133/8895)	1.06 (0.98 - 1.15)	0.17
Evening (17:00 – 23:00)	40.3% (889/2205)	1.35 (1.20 - 1.52)	<0.001
**Day of week**			
Weekday call	35.4% (5637/15918)	Reference	
Saturday call	50.0% (44/88)	1.68 (1.06 – 2.66)	0.03
Sunday call	45.4% (39/86)	1.52 (0.95 – 2.42)	0.08
**Gender of research assistant caller**			
Male caller	29.6% (444/1500)	Reference	
Female caller	36.2% (5276/14592)	1.21 (1.06 - 1.39)	0.01

We have recruitment data on 4123 individuals (3169 eligible referrals, 954 ineligible referrals based on study inclusion and exclusion criteria). Of 3169 eligible individuals, 982 individuals (31%) were successfully recruited. Table 3 provides the demographic differences between individuals recruited and non-recruited individuals among those referred. Amongst those recruited, we noted significant differences in recruitment success and participant demographics by recruitment method.

**Table 3:** General recruitment results for all methods and all studies participating in the CRI *

	Recruited	Not recruited	p-value	
	n=1003	n=2248	Univariate	Multivariate**
Mean age (95% CI)	30.88	29.98	<0.001	0.003
	(30.56 - 31.19)	(29.61 - 30.35)		
	n=841	n=673		
Mean income quintile (95% CI)	3.20	3.11	0.41	0.11
	(3.11 - 3.30)	( 3.04 - 3.17)		
	n=793	n=1910		
Proportion from the general Canadian population	83.1%	80.3%	0.40	0.40
	(152/183)	(451/562)		
Proportion with gestational age >= 24 weeks	44.7%	61.7%	<0.001	0.03
	(441/986)	(1807/2927)		
Mean number of calls	4	4	0.01	0.04
Median between quartiles 1 and 3	(2.0 – 6.0)	(2.0 – 8.0)		
	n=286	n=745		

*Results exclude those 960 individuals not eligible for recruitment based on the assigned study inclusion or exclusion criteria.

**Controlling for differences between recruiting study.

### Recruitment methods

Results for each recruitment method are presented in the following order 1) any significant demographic differences in referrals compared to fax referrals, 2) any significant difference in recruitment rate compared to fax referrals, 3) any significant demographic difference in recruits compared to fax referrals. For all studies, there was no significant difference in the mean age between recruitment methods for referrals or recruits (Table 4). Amongst CHILD Study recruits, there were no significant difference in the recruit demographics between any of the recruitment methods for maternal mean age, maternal attendance at a post-secondary institution, maternal smoking history and paternal history of asthma (Table 5).

**Table 4:** **Demographics of referrals and recruits by recruitment method for all studies**

	Fax	In-clinic	Tradeshows	Direct	Media (Free and paid)
	(Reference)				
**Referrals**					
Mean age (SD)	30.2 (5.0)	30.4 (4.8)	30.8 (4.6)	33.0 (5.2)	31.0 (4.9)
	n = 1888	n = 480	n = 114	n = 14	n = 45
Above 24 weeks GA	57.98%	55.04%	60.83%	51.22%	14.68%*
	(1144/1973)	(879/1597)	(368/605)	(21/41)	(43/293)
Mean income quintile (SD)	3.3 (1.4)	2.9* (1.4)	3.2 (1.3)	2.3* (1.5)	3.2 (1.4)
	n = 2044	n = 1479	n = 531	n = 7	n = 58
**Recruits**					
Mean age (SD)	30.9 (4.5)	30.7 (4.7)	31.0 (4.4)	33.3 (5.5)	31.2 (4.9)
	n = 301	n = 388	n = 91	n = 12	n = 42
Above 24 weeks GA	45.36%	54.39%*	54.95%	68.75%	11.25%*
	(137/302)	(217/399)	(50/91)	(11/16)	(18/160)
Mean income quintile (SD)	3.5 (1.3)	3.0* (1.4)	3.2 (1.3)	n < 5	3.4 (1.5)
	n = 290	n = 366	n = 79		n = 36

*p ≤ 0.05 All analysis are adjusting for recruiting study.

Income Quintiles range from 1 - 5.

**Table 5:** **Demographics of recruits by recruitment method among CHILD study participants**

	Fax	Tradeshow	Direct	In-clinic	Paid-media	Free-media
	(Reference)					
**Maternal characteristics**						
Mean age (SD)	31.0 (4.3)	31.2 (4.3)	33.3 (5.5)	30.8 (4.7)	30.3 (4.9)	32.4 (5.0)
	n = 267	n = 90	n = 12	n = 387	n = 26	n = 14
Above 24 weeks GA	47.43%	54.95%	68.75%	54.64%*	26.92%*	35.29%*
	(129/272)	(50/91)	(11/16)	(212/388)	(7/26)	(6/17)
Mother Caucasian	85.90%	85.14%	90.00%	78.18%*	82.35%	88.24%
	(195/227)	(63/74)	(9/10)	(240/307)	(14/17)	(15/17)
Mother attended post-secondary	92.63%	91.78%	90.00%	89.78%	94.44%	82.35%
	(176/190)	(67/73)	(9/10)	(281/313)	(17/18)	(14/17)
Mother has or had asthma	20.26%	22.97%	30.0%	23.45%	23.53%	47.06%*
	(46/227)	(17/74)	(3/10)	(72/307)	(4/17)	(8/17)
Mother smokes daily or occasionally	6.84%	1.35%	0.00%	4.23%	17.65%	5.88%
	(13/190)	(1/74)	(0/10)	(13/307)	(3/17)	(1/17)
**Paternal characteristics**						
Father born in Canada	89.23%	74.19%*	100.00%	79.75%*	88.89%	80.00%
	(116/130)	(23/31)	(6/6)	(126/158)	(8/9)	(8/10)
Father attended post-secondary	86.26%	90.32%	100.00%	78.48%	88.89%	90.00%
	(113/131)	(28/31)	(6/6)	(124/158)	(8/9)	(9/10)
Father has or had asthma	26.92%	25.81%	33.33%	20.89%	22.22%	10.00%
	(35/130)	(8/31)	(2/6)	(33/158)	(2/9)	(1/10)
**Family characteristic**						
Family income ≥ $40,000	95.73%	95.65%	90.00%	88.77%*	94.44%	93.33%
	(202/211)	(66/69)	(9/10)	(253/285)	(17/18)	(14/15)
Married/common law	97.70%	92.31%*	100.00%	93.39%*	88.00%*	88.24%
	(255/261)	(84/91)	(16/16)	(353/378)	(22/25)	(15/17)

*p ≤ 0.05.

#### In-clinic

In-clinic referrals had a significantly lower mean income quintile (2.9, SD 1.4; n = 1479) compared to fax referrals (3.3, SD = 1.4; n = 2044; p < 0.05 for both). In-clinic referrals continued to have a lower mean income quintile (Table 6) when analyses were restricted to clinics where both fax and in-clinic recruitment methods were used.

**Table 6:** **Demographics of referrals and recruits by recruitment method among locations that participated in more than 1 recruitment method**

	Fax	In-clinic
**Referrals**		
Mean age (SD)	29.4 (5.3)	29.9 (4.9)
	n = 290	n = 175
Above 24 weeks GA	18.2%	48.9%*
	(45/248)	(274/560)
Mean income quintile (SD)	2.8 (1.4)	2.6* (1.4)
	n = 272	n = 535
**Recruits**		
Mean age (SD)	29.76 (4.26)	30.20 (4.62)
	n = 27	n = 128
Above 24 weeks GA	11.54%	56.49%*
	(3/26)	(74/131)
Mean income quintile (SD)	3.1 (1.5)	2.8 (1.3)
	n = 26	n = 119

Income Quintiles range from 1 - 5.

*p-value<0.05.

Among CHILD study recruits, there was a significant difference in the proportion of married individuals between the fax and in-clinic recruits (Fax: 97.7% married; In-Clinic: 93.4% married; p < 0.05). The proportion of individuals who identified themselves as Caucasians differed between the fax and in-clinic recruits (Fax: 85.9%, In-Clinic: 78.2%, p < 0.05). For all studies, compared to fax recruits, in-clinic recruits were later in pregnancy (Fax: 45.4% ≥ 24 weeks GA; In-Clinic: 54.4% ≥ 24 weeks GA; p < 0.05) and had a significantly lower mean income quintile (Fax: 3.5/5, SD 1.3; n = 290, In-Clinic: 3.0/5, SD 1.4; n = 367; p < 0.05). In-clinic recruits continued to be later in GA and have a lower mean income quintile when analyses were restricted to clinics where both fax and in-clinic recruitment methods were used.

#### Tradeshow

Recruitment success from tradeshows depended on whether a raffle was held. Compared to fax recruits, when a raffle was held, referrals were 44% less likely to be recruited (OR 0.56, 95% CI 0.41, 0.75; p < 0.001; Table 7). When a raffle was not held, referrals were 72% less likely to be recruited compared to fax recruits (OR 0.28, 95% CI 0.14, 0.58, p < 0.001). Analyses controlled for differences between studies, GA at the time of referral, and family income quintiles.

**Table 7:** Odds ratio (OR) of recruiting a pregnant woman by recruitment method* (n = 2685)

	Number of recruits	Odds ratio	p-value
		[95% CI]	
Fax	28%	Reference	
	(308/1112)		
Direct	43%	1.93	0.40
	(17/40)	(0.41 - 9.01)	
In-clinic	31%	1.02	0.81
	(399/1291)	(0.84 - 1.25)	
Media			
Free-media	68.0%	12.99	<0.001
	(77/114)	(4.18 - 40.43)	
Paid-media	72.0%	4.63	<0.001
	(86/120)	(2.08 - 10.31)	
Tradeshows			
No raffle was held	21%	0.56	<0.001
	(82/398)	(0.41 - 0.75)	
A raffle was held	12%	0.28	<0.001
	(11/92)	(0.14 - 0.58)	

Goodness of fit (p-value) = 0.79 (not significant indicating good fit).

*Analysis controlled for recruiting study, gestational age and family income quintile.

#### Media (free and paid media)

Referrals from media were earlier in pregnancy (14.7% of women >24 weeks GA) compared fax referrals (58.0% >24 weeks GA; p < 0.05). Free-media referrals were 13 times more likely to be recruited than fax referrals (OR 13.0, 95% CI 4.18, 40.43: p < 0.001), while paid-media referrals were over 4 times more likely to be recruited than fax referrals (OR 4.6, 95% CI 2.08, 10.31; p < 0.001).

For all study recruits, women recruited through media were earlier in pregnancy (11.3% of women >24 weeks GA) compared to fax recruits (45.4% >24 weeks GA; p < 0.05). This statistically significant difference was similar in CHILD study recruits. For CHILD study recruits, media recruits were earlier in pregnancy (Free-media: 35.3% of women >24 weeks GA; Paid-media: 26.9% >24 weeks GA) compared to fax (47.4%, p < 0.05 for both). Among CHILD Study recruits, free-media recruits were more likely to report a history of asthma compared to fax recruits (Free-media: 47.1% versus Fax: 20.3%; p < 0.05). There was no difference in maternal asthma history between paid-media and fax recruits.

### Comparison of CHILD Study Recruits to a Reference Population

CHILD study participants recruited from fax and in-clinic were different from each other and from the reference population. CHILD study recruits, overall, were more likely to attend university (Total: 90.9%, Fax: 92.6%, in-clinic 89.8%; Table 8) versus the reference population (52.0%; p < 0.05 for all three); were less likely to smoke (Total: 5.0%, Fax: 6.8%, In-clinic 4.2%) versus reference (18.6%; p < 0.05 for all three); and come from the highest income quartile (Total: 81.6%, fax: 83.9%; in-clinic: 77.9%) versus reference population (42.0%; p < 0.05).

**Table 8:** **Comparison of CHILD study recruits, by recruitment method, versus a reference population**

		Reference population	Recruits		
			Total	Fax	In-clinic
			n = 836	n = 308	n = 399
Married or common law**		93.5%	94.56%	97.7%*	93.4%
		(1895/2026)	(747/790)	(255/261)	(353/378)
Attended university (yes/no)		52.0%	90.9%*	92.6%*	89.8%*
		(1049/2018)	(566/623)	(176/190)	(281/32)
Current daily or occasionally cigarette smoking		18.6%	5.0%*	6.8%*	4.2%*
		(379/2041)	(31/617)	(13/177)	(13/294)
**Ethnic or racial group**	Caucasian**	77.5%	82.3%*	85.9%*	78.2%
		(1531/1986)	(538/654)	(195/227)	(240/307)
	Asian	9.2%	8.9%	6.7%	11.4%
		(182/1986)	(55/621)	(13/194)	(35/307)
	East Indian	3.1%	0.8%*	1.3%	0.3%*
		(62/1986)	(5/654)	(3/227)	(1/307)
	Black	1.7%	3.2%*	1.8%	3.9%*
		(33/1986)	(21/654)	(4/227)	(12/307)
	First Nations	3.7%	6.1%*	3.5%	6.8%*
		(71/1986)	(40/654)	(8/227)	(21/307)
**Household income quartiles**	Less than $12,000	9.7%	2.6%	0.9%	3.2%
	*Less than $19 999*	(176/1816)	(16/610)	(2/211)	(9/285)
Top: reference cut off	$12,000 to $24,999	15.9%	5.1%	3.3%	8.1%
	*$20,000 to $39,999*	(288/1816)	(31/610)	(7/211)	(23/285)
	$25,000 to $49,999	32.5%	10.7%	11.8%	10.9%
	*$40,000 to $59,999*	590/1816	65/610	25/211	31/285
Bottom/italicized: CHILD cut off	$50,000 or more**	42%	81.6%*	83.9%*	77.9%
	*$60,000 or more*	(762/1816)	(498/610)	(177/211)	(222/285)
**Medical conditions**	High blood pressure	3.6%	1.4%*	0.4%	2.3%
		(73/2014)	(9/655)	(1/228)	(7/307)
	High cholesterol	3.6%	2.0%	1.8%	2.6%
		(73/2016)	(13/655)	(4/224)	(8/307)
	Diabetes	2.3%	2.6%	3.1%	3.3%
		(46/2013)	(17/655)	(7/221)	(10/307)
	Kidney disease	1.0%	0.9%	0.9%	1.0%
		(20/2013)	(6/654)	(2/226)	(3/307)
	Heart disease	0.7%	0%*	0%	0%
		(14/2012)	(0/639)	(0/229)	(0/307)

*p < 0.05 compared to reference population.

**p < 0.05 between fax and in-clinic recruits.

Although the proportion of married individuals among total recruits (94.6%) and the reference population was not statistically significantly different (93.5%; p < 0.05), there were significant differences in marital status between fax recruits and the reference population (97.7% married; p < 0.05). This difference from the reference population was not observed among in-clinic recruits (93.4%; p > 0.05). While total recruits and fax recruits, were more likely to be Caucasian (Total: 82.3%; Fax: 85.9% versus reference 77.5%; p < 0.05 for both), the proportion of Caucasian amongst in-clinic recruits was not significantly different from the reference population (78.2%; p > 0.05). For the recruits that identified themselves of first-nations origin, there were no significant difference between the fax recruits (3.5% identifying themselves as first-nation) compared to the reference population (3.7%, p > 0.05). However, in-clinic recruits has a disproportionate percentage of individuals of first nations origin (6.8%; p < 0.05) when compared to the reference population.

### Advertising RCT

For the RCT analysis, inclusion of referrals 2 days after the advertisement intervention provided the best model fit. Between the free and paid media recruitment methods, referrals differed in mean age, proportion of women >24 weeks GA, and mean income quintile (Table 9). Amongst all paid-media referrals, internet referrals were the oldest (Internet: mean age 31.3 yr., SD 4.3); paid-to-read referrals had the highest proportion of women later in pregnancy (76.2% >24 weeks GA); and transit advertisements had the highest mean income quintile (mean 3.4, SD 1.4). The demographic findings by paid media method were all statistically different compared to free-media referrals (mean age: 30.6 yr, SD 4.8; 45% >24 weeks GA; mean income quintile: 2.8, SD 1.4; all three p < 0.05).

**Table 9:** **Demographics for referrals for media (free and paid)**

	Free-media	Paid media							
		Postal	Free-to-read	Transit	Internet	Radio	Trade specific publication	Paid-to-read	Birth issues
	n = 41	n = 17	n = 197	n = 230	n = 430	n = 207	n = 1107	n = 43	n = 1712
Mean age (SD)	30.6 (4.8)	29.9 (3.8)	30.8 (5.1)	31.1 (4.3)	31.3 (4.3) *	30.2 (4.5)	30.7 (4.6)	30.4 (3.6)	30.5 (4.6)
	n = 13	n = 10	n = 44	n = 83	n = 147	n = 63	n = 379	n = 19	n = 589
Above 24 weeks GA	45.00%	41.18%	52.97%	52.34%	56.35%	44.22%*	55.88%	76.19%*	55.66%
	n = 18	n = 7	n = 98	n = 112	n = 235	n = 88	n = 594	n = 32	n = 915
Mean income quintile (SD)	2.8 (1.4)	2.9 (1.5)	3.4 (1.3)	3.4* (1.4)	3.1 (1.4)	3.0 (1.4)	3.1 (1.4)	3.1 (1.4)	3.1 (1.4)
	n = 32	n = 17	n = 152	n = 193	n = 372	n = 159	n = 939	n = 42	n = 1466

*p < 0.05.

Free-to-read print (e.g. See, Metro) was the most effective method of advertising (OR 3.3, 95% CI 2.34, 4.51; p < 0.05; Table 10) with a cost of $63.44/recruit. The most cost-effective method of recruitment, while still providing a significant recruitment advantage over no-advertisement (reference) was advertising in Birth Issues at a cost of $1.56/recruit (OR 1.97, SD 1.64. 2.37; p < 0.05). Each referral costs the study human resources in an attempt to convert the referral to a recruit. $/OR measurements accounts for recruitment effectiveness (converting a referral into a recruit) whereas a $/recruit measurement only accounts for recruits. A recruitment method that has higher recruit:referral, as captured by an OR, is a more cost effective recruitment method. Based on this metric, Birth Issues was still the most efficient paid-media method ($0.79/OR of recruiting someone), followed by trade-specific publications ($13.27), Internet ($15.00), and free-to-read print ($19.52).

**Table 10:** **Media (free and paid) recruitment method on number of referrals and number of recruits ordered from best dollar for value to most expensive dollar value**

		Total						Media specific		
		# of days	Number of recruits	Recruits/day	$/recruit	Odds ratio (95% CI) of recruiting someone	$/OR of recruiting someone	Number of recruits	Recruits/day	$/recruit
Free-media		11	10	0.91	0	0.66 (0.35 - 1.26)	0	3	0.27	0
Paid-media										
	Birth Issues	367	514	1.40	$1.56	1.97* (1.64 - 2.37)	$0.79	56	0.15	$14.29
	Trade-specific publication	225	338	1.50	$16.59	1.25* (1.04 - 1.50)	$13.27	39	0.17	$143.77
	Internet	77	132	1.71	$22.35	1.49* (1.21 - 1.84)	$15.00	7	0.09	$421.43
	Free –to-read print	18	47	2.61	$63.44	3.25* (2.34 - 4.51)	$19.52	21	1.17	$141.98
	Radio	53	53	1.00	$108.68	1.51* (1.13 - 2.03)	$71.97	8	0.15	$720.00
	Paid–to-read print	6	19	3.17	$267.37	1.92* (1.21 - 3.07)	$139.26	2	0.33	$2540.00
	Transit	69	74	1.07	$31.08	1.15 (0.89 - 1.48)	$27.03	18	0.26	$127.78
	Postal	3	4	1.33	$112.87	1.57 (0.58 - 4.20)	$71.89	0	0.00	N/A
Mon-Thurs. vs. Fri						1.97* (1.61 - 2.41)				
Sat-Sun vs. Fri						0.24* (0.17 - 0.33)				
Low season vs. high season						0.59* (0.49 - 0.71)				

Low season is defined as July, August and December.

Autocorrelation of 2 days is considered in the model. A model of 1,3, and 4 days was tested. The 2 day model was provided the best model fit.

*p < 0.05.

Among paid advertisements with a recruitment rate significantly greater than no advertisement (reference), radio and Birth Issues had the slowest recruitment rate averaging only 1.0 recruits/day and 1.4 recruits/day respectively. Both free-to-read and paid-to-read print had a greater number of recruits per day in total than the other paid-media methods (Free-to-read: 2.61 recruits/day; Paid-to-read: 3.17 recruits/day).

Several demographic differences were noted among the paid and free-media recruits (reference). Recruits from free-media were less likely to have post-secondary education (55.6%) versus recruits from paid-media (range of 89.7% to 100% with post-secondary education; p < 0.05; Table 11). Similarly, free-media recruits were less likely to have a family income ≥ $80,000 (33.3%) versus paid-media recruits (range of 60.7% to 77.3%; p < 0.05). Finally, free-media recruits were less likely to be married (77.8%) when compared to paid-media recruits (Range: 93.6% to 100%; p < 0.05).

**Table 11:** **Demographics by recruits by media (paid and free) recruitment**

	Free-media (Reference)	Paid-media							
		Postal	Free-to-read print	Transit	Internet	Radio	Trade-specific publications	Paid–to-read print	Birth issues
**Maternal characteristics**									
Mean Age (SD)	31.5 (4.6)	30.1 (6.0)	32.3 (4.4)*	31.2 (3.8)	31.5 (4.3)*	30.7 (4.4)	30.9 (4.5)*	30.4 (3.7)	30.9 (4.5)*
	n = 10	n = 4	n = 27	n = 58	n = 126	n = 50	n = 313	n = 18	n = 479
Above 24 weeks GA	30.00%	25.00%	51.85%	53.45%**	53.54%*	40.00%	55.27%*	72.22%*	53.75%*
	(3/10)	(1/4)	(14/27)	(31/58)	(68/127)	(20/50)	(173/313)	(13/18)	(258/480)
Mother Caucasian	62.50%	100.00%	95.83%	90.57%	76.64%	85.71%	82.68%	81.25%	81.28%
	(5/8)	(4/4)	(23/24)	(48/53)	(82/107)	(24/28)	(191/231)	(13/16)	(291/358)
Mother attended post-secondary	55.56%*	100.00%	100.00%	92.59%	90.00%	93.10%	89.79%	100.00%	89.70%
	(5/9)	(3/3)	(24/24)	(50/54)	(99/110)	(27/29)	(211/235)	(18/18)	(331/369)
Mother reports no current or prior health conditions	25.00%	25.00%	12.50%**	24.53%	24.30%	21.43%	22.08%*	12.50%	22.91%*
	(2/8)	(1/4)	(3/24)	(13/53)	(26/107)	(6/28)	(51/231)	(2/16)	(82/358)
Mother has or had asthma	25.00%	0.00%	8.33%**	18.87%	16.82%	32.14%	19.91%	6.25%	21.79%
	(2/8)	(0/4)	(2/24)	(10/53)	(18/107)	(9/28)	(46/231)	(1/16)	(78/358)
Mother smokes daily or occasionally	0.00%	0.00%	0.00%	5.66%	5.61%	3.57%	6.06%	6.25%	5.03%
	(0/8)	(0/4)	(0/24)	(3/53)	(6/107)	(1/28)	(14/231)	(1/16)	(18/358)
**Paternal characteristics**									
Father born in Canada	66.67%	30.00%	91.67%	85.71%	86.79%	75.00%	85.44%	33.33%**	83.33%
	(2/3)	(3/10)	(11/12)	(24/28)	(46/53)	(12/16)	(88/103)	(1/3)	(145/174)
Father attended post-secondary	66.67%	66.67%	75.00%	85.71%	88.68%	81.25%	82.52%	100%	83.91%
	(2/3)	(2/3)	(9/12)	(24/28)	(47/53)	(13/16)	(85/103)	(3/3)	(146/174)
Father has or had asthma	33.33%	33.33%	33.33%	28.57%	16.98%	37.50%	18.45%	0.00%	24.14%
	(1/3)	(1/3)	(4/12)	(8/28)	(9/53)	(6/16)	(19/103)	(0/3)	(42/174)
**Family characteristics**									
Family income ≥ $80,000	33.33%*	66.67%	77.27%	75.00%	68.93%	60.71%	68.66%	75.00%	66.76%
	(3/9)	(2/3)	(17/22)	(39/52)	(71/103)	(17/28)	(149/217)	(12/16)	(231/346)
Family income ≥ $40,000	77.78%	66.67%	95.45%	96.15%	93.20%	89.29%	94.93%	100%	92.49%
	(7/9)	(2/3)	(21/22)	(50/52)	(96/103)	(25/28)	(206/217)	(16/16)	(320/346)
Married /Common law	77.78%*	100.00%	100.00%	97.74%	95.12%	95.83%	93.79%	100.00%	93.56%
	(7/9)	(4/4)	(27/27)	(54/57)	(117/123)	(46/48)	(287/306)	(17/17)	(436/466)

Analyses controlled for the number of concurrent advertisements active.

*p ≤ 0.05 ; **p ≤ 0.08.

When we examined specific demographic differences within paid media, free-to-read print recruits were older (mean age 32.3 years, SD 4.4) while recruits from trade-specific publications (30.9 years, SD 4.5) and Birth Issues (30.9 years, SD 4.5) were younger than free-media recruits (31.5 years, SD 4.6; p < 0.05 for all 3). Free-media recruits were earlier in pregnancy (30.0% >24 weeks GA) compared to most of the paid-media advertisements including the trade-specific publications (55.3% >24 weeks GA; p < 0.05), Birth Issues (53.8%; p < 0.05) and paid-to-read print (72.2%; p < 0.05). Recruits from the trade-specific publications and Birth Issues were less likely to report a prior health condition (22.1% and 22.9% respectively) versus free-media recruits (25.0%; p < 0.05 for both).

## Discussion

Our study identifies that recruitment methods can introduce participant demographic bias, which expands on our understanding that unrepresentative recruitment can result in selection biases [16]. This analysis of recruitment methods for longitudinal pregnancy cohort studies found substantial differences in the recruitment rates between recruitment methods. Participant demographic bias may be due to clinical, social, ethical and ethnic differences [17–19]. Results from the CRI suggest that 1) demographic differences in the individuals referred by each method leads to 2) demographic differences in the individuals recruited by each method which results in, 3) demographic differences in the individuals recruited when compared to a reference population. This trend is most obvious when examining demographic differences in mean income from referrals through to recruits. Multiple recruitment methods are required to overcome this source of participant demographic bias.

Demographic differences in referrals between recruitment methods likely represent a combination of factors including differences in clinic demographics, referral bias, and the level of engagement from front-line clinic staff. Physicians participating in the fax recruitment method present the study information to those patients who they feel would be most likely to join the study with a possible bias to married, higher socio-economic status individuals. The in-clinic recruiters approach all individuals and present the benefits of participating; this presentation may appeal to a wider population. This bias in study presentation is reflected in a lower mean family income and more variable marital status for in-clinic referrals and, consequently, in-clinic recruits. Demographic differences by recruitment method highlight the importance of using multiple recruitment strategies to recruit a population-representative sample.

Recruitment methods that encourage prospective participants to contact our central recruitment office, such as free and paid-media strategies, save RA calls and RA time. Our study showed that respondents to both type of media had the highest rate of recruitment. However, referrals (and recruits) from free-media respondents were more likely to have a health condition associated with the research (e.g. asthma for the CHILD study). This health-condition bias was not observed among the paid-media recruits and, in general, there were few substantive demographic differences between paid-media and fax recruits (reference). The less expensive advertisement methods have a lower recruit/day rate and conversely, the more expensive advertisement methods provide a higher number of recruits/day. Free and paid-media are effective methods of recruiting participants, although a study’s choice of paid-media advertisement will be determined by the study’s budget and recruitment time frame.

Recruiting a sample of pregnant women has required the development of substantial recruitment infrastructure with support from multiple stakeholders. We worked with all four birth cohort local principal investigators, four hospitals, two health regions, multiple physicians (both academic-appointed and those in private-practice), multi-disciplinary healthcare providers (e.g. mid-wives, nurses), health-care groups (laboratory services, ultrasound clinics), and community organizations (baby stores, media organizations, trade show organizers, interest groups) in developing the CRI. Support from all stakeholders is a pre-requisite to developing and using multiple recruitment strategies.

We developed a CRI referral process that met the needs of community-based clinicians. Lack of support from clinicians can present challenges to recruitment [20]. Barriers to staff participation in research include: time [15, 21], understanding the many complicated protocols [22], a lack of perceived rewards [21], and interference with the health-care professional/patient relationship [23, 24]. The CRI attempted to overcome many of these barriers. Health-care professionals do not give detailed study explanations nor do they consent participants; this reduces the recruitment burden on their practice with minimal impact on their patient relationship [21, 23]. Distributing prospective research participants across several studies broadens the entry criteria making it more efficient for healthcare professionals to participate in numerous studies with varying inclusion and exclusion criteria. The $250 reimbursement was based on evidence supporting monetary incentives to encourage healthcare professionals to recruit patients [25]. As a result of our work, the fax process provided the greatest number of referrals/day compared to all other recruitment methods including sitting in-clinic.

One of the limitations of this study is the limited demographic data available for all referrals. The choice of information collected from referrals was focused on obtaining the least amount of information necessary to efficiently triage patients to the participating CRI studies. As an example, while some studies excluded individuals on anti-depressant medication, we elected not to put this criterion on the fax recruitment sheet as it would increase the burden for the community-based obstetricians. Similarly, the in-clinic recruiter did not routinely obtain the referral’s date of birth or marital status since this information was not critical for the distribution of prospective participants to participating research studies. The use of postal codes as a surrogate for household income is a result of the necessarily limited data collected on referrals. Although we do not have any detailed recruit demographic data for the non-CHILD studies, we used more detailed demographic data from the CHILD study (over 85% of all CRI recruits) in an attempt to overcome this limitation.

We encountered several difficulties in designing and implementing the advertisement RCT. We had originally proposed to run a single advertisement intervention (i.e. only transit or only radio or only free-to-read print). However, we could not identify any prior publications that identified the most effective advertisement method for health research study recruitment. As a result, we chose to test common advertisement methods. This decision resulted in reduced power when examining the effect of a single advertisement intervention on recruitment (a more definitive study). Some of our media outlet vendors did not run our advertisement, while others did not run the advertisement during the proscribed period and compensated us, without telling us, by running the advertisement at other times or for a longer period of time. We tried to control for this “contamination” through statistical analyses by controlling for the number of studies run on a specific day.

We chose to implement the advertisement RCT within the context of the CHILD study due to fiscal and staffing considerations. Using the CHILD study as the recruiting study resulted in compromises to the advertisement RCT. We originally proposed to test each advertisement intervention twice in order to limit any seasonal bias on referrals and recruits. Unfortunately, the CHILD Study closed recruitment prior to the completion of two advertisement “runs” for each advertisement intervention. We tried to control for the seasonal effect of advertisement in the regression analysis in an attempt to control for this limitation.

One of the strengths of this analysis is the large number of referrals (over 5000). The CRI was implemented at all 4 obstetrical hospitals in Edmonton to ensure a sample of pregnant women that was as population representative as possible. The prospective, systematic capture of referral data ensures minimal recall bias. The systematic, real-time, capture of recruit demographics could be utilized to adjust recruitment strategies to ensure a population-representative sample. We believe the results obtained from this analysis are timely and generalizable to currently recruiting pregnancy and birth cohort studies.

We completed over 16,000 telephone calls as part of the CRI resulting in a significant human resource cost. Future research may examine the success of volunteers, automated phone dialers and/or call centers in recruiting study participants. Future research may also include conducting multiple RCTs of each of the paid advertisement versus a non-advertisement control outside the confines of an active research study to better understand the effectiveness of each of the advertisement methods in medical research.

## Conclusion

Successful recruitment requires collaboration and active engagement from all major stakeholders. Implementing a study of recruitment methods into large, population-based studies requires pre-planning and additional study infrastructure. The results of our study help improve our understanding one of the most expensive and time-consuming but often over-looked aspects of study design. A variety of recruitment methods are required to generate a large, population-representative birth-cohort study.
